# Meta-analysis of variance in tDCS effects on response inhibition

**DOI:** 10.1038/s41598-024-70065-7

**Published:** 2024-08-19

**Authors:** Luca Lasogga, Chiara Gramegna, Dario Müller, Ute Habel, David M. A. Mehler, Ruben C. Gur, Carmen Weidler

**Affiliations:** 1 Department of Psychiatry, Psychotherapy and Psychosomatics, Faculty of Medicine, RWTH Aachen, Pauwelsstraße 30, 52074 Aachen, North Rhine-Westphalia Germany; 2 PhD Program in Neuroscience, School of Medicine and Surgery, University of Milano-Bicocca, Monza, Italy; 3 Institute of Neuroscience and Medicine, JARA-Institute Brain Structure Function Relationship (INM 10), Research Center Jülich, Wilhelm-Johnen-Straße, 52438 Jülich, Germany; 4 Institute for Translational Psychiatry, University of Münster, 48149 Münster, Germany; 5 Cardiff University Brain Research Imaging Centre (CUBRIC), School of Psychology, Cardiff University, Cardiff, CF24 4HQ UK; 6 Brain Behavior Laboratories, Department of Psychiatry, University of Pennsylvania Perelman School of Medicine, Philadelphia, USA; 7 Office 117, Wendlingweg 2, 52074 Aachen, Germany

**Keywords:** Response inhibition, Transcranial direct current stimulation, Coefficient of variation ratio, Inter-individual differences, Cognitive neuroscience, Cognitive control

## Abstract

Deficiencies in response inhibition are associated with numerous mental health conditions, warranting innovative treatments. Transcranial direct current stimulation (tDCS), a non-invasive brain stimulation technique, modulates cortical excitability and has shown promise in improving response inhibition. However, tDCS effects on response inhibition often yield contradictory findings. Previous research emphasized the importance of inter-individual factors that are mostly ignored in conventional meta-analyses of mean effects. We aimed to fill this gap and promote the complementary use of the coefficient of variation ratio and standardized mean effects. The systematic literature search included single-session and sham-controlled tDCS studies utilizing stop-signal task or Go-NoGo tasks, analyzing 88 effect sizes from 53 studies. Considering the impact of inter-individual factors, we hypothesized that variances increase in the active versus sham tDCS. However, the results showed that variances between both groups did not differ. Additionally, analyzing standardized mean effects supported previous research showing an improvement in the stop-signal task but not in the Go-NoGo task following active tDCS. These findings suggest that inter-individual differences do not increase variances in response inhibition, implying that the heterogeneity cannot be attributed to higher variance in response inhibition during and after active tDCS. Furthermore, methodological considerations are crucial for tDCS efficacy.

## Introduction

The ability to withhold one’s responses is a crucial skill in a wide range of activities such as decision-making, emotional regulation and behavioral control. This form of impulsivity, called response inhibition, is associated with higher risk-taking behavior^1^ and emotional dysregulation^2^. Response inhibition is defined as the ability to suppress motor, cognitive and affective functions resulting in undesirable behavior^3,4^. Both impulsivity and response inhibition are associated with a variety of mental disorders including substance use disorder, attention-deficit/hyperactivity disorder (ADHD), obsessive compulsive disorder (OCD), eating disorders, and psychopathy^5–10^. Deficiencies in impulsivity and response inhibition are linked to functional brain alterations such as decreased activation in the left prefrontal cortex (PFC) and the right inferior frontal gyrus (rIFG)^11,12^.

To address PFC related deficits in response inhibition, non-invasive brain stimulation (NIBS) has become increasingly relevant in the past 20 years. Among a variety of NIBS methods, transcranial direct current stimulation (tDCS) emerged as a cost-effective method. TDCS delivers weak electrical currents to cortical regions and neuronal networks, modifying the resting membrane potential of neurons. Theoretically, anodal stimulation increases resting membrane potential, while cathodal stimulation decreases it^13^. Yet, an increasing amount of research questions this dichotomous view^14^.

Thus far, research on tDCS effects on response inhibition has produced heterogeneous results. Whereas some studies reported beneficial tDCS effect on response inhibition^15,16^, others reported no effects at all^17,18^. Meta-analyses revealed an overall benefit of tDCS on response inhibition, although effect sizes were small^19,20^. Despite a small effect, inconsistencies between studies may be explained by inter-individual differences. Vergallito et al.^21^ identified stable and variable factors contributing to inter-individual variability. Among stable factors are genotypes, morphological disparities, and biological sex, which may play a significant role in explaining differences in tDCS responses between individuals. Notably, genes encoding brain-derived neurotrophic factors and Catechol-O-Methyltransferase have been identified to interact with tDCS effects^22–24^. Additionally, morphological disparities such as skull thickness and composition^21,25^ may be associated with tDCS efficacy. Age and biological sex, factors related to skull thickness, have also been suggested to modulate tDCS effects^21,26,27^. Moreover, recent evidence indicates that cortical thickness^28^ and electric field magnitude^29^ can influence the outcomes of tDCS interventions. Variable factors such as mental disorder may also influence tDCS effectiveness. Patients with schizophrenia, Alzheimer’s disease, major depressive disorder (MDD), and Parkinson’s disease react differently to tDCS compared to healthy individuals^30^. MRI research indicated that electric field strength was significantly diminished in patients (Schizophrenia and MDD) compared to healthy controls^31^. Substance use, for instance nicotine consumption, may also influence tDCS efficacy^32,33^. This is supported by findings suggesting that nicotine intake can cancel out tDCS effects of long-term plasticity^34^.

Conventional meta-analyses have predominantly focused on standardized mean effects^35^, often not addressing inter-individual differences. Homan et al.^36^ were the first to investigate inter-individual variation in NIBS research and found increased variance in active compared to sham tDCS in patients with schizophrenia. In contrast to previous meta-analyses focusing on mean effect differences of tDCS by calculating Cohens d or Hedges g^20,37^, this meta-analysis aimed to investigate the heterogeneity—or difference in variance—between active and sham tDCS conditions. Comparing individual response differences necessitates the calculation of a ratio between active and sham variance in response to a specific treatment^35^. The logarithm of the coefficient of variation ratio (lnCVR) is a metric that can measure whether treatment groups show higher variation compared to placebo groups^35^. This statistic, employed to compare variability between groups, has been used for other treatment approaches such as dietary restrictions^38^ and schizophrenia medication^39^.

The rationale behind this method is that if variance in response inhibition is higher in active compared to sham tDCS, inter-individual differences might influence the effect of tDCS. If individual factors play a role in the effectiveness of tDCS, we expect increased variation in response to active compared to sham tDCS. The primary objective is to use the lnCVR to compare variances between active and sham tDCS and incorporate standard tDCS parameters into the analysis to investigate their individual contribution to variability in tDCS groups.

In addition to assessing variance differences, we examine mean differences in response inhibition performance between active and sham tDCS to replicate previous findings^20^. Accordingly, we expect to find a positive effect of active tDCS on response inhibition, and moderating effects of tDCS parameters—including target electrode position and task. Measuring variance differences between active and sham tDCS and incorporating tDCS parameters may inform the development of tDCS protocols that effectively modulate response inhibition. In addition, comparing mean differences and adding more recent studies may allow us to replicate findings^20^ and determine the robustness of previously reported effects.

## Results

**Figure 1:**
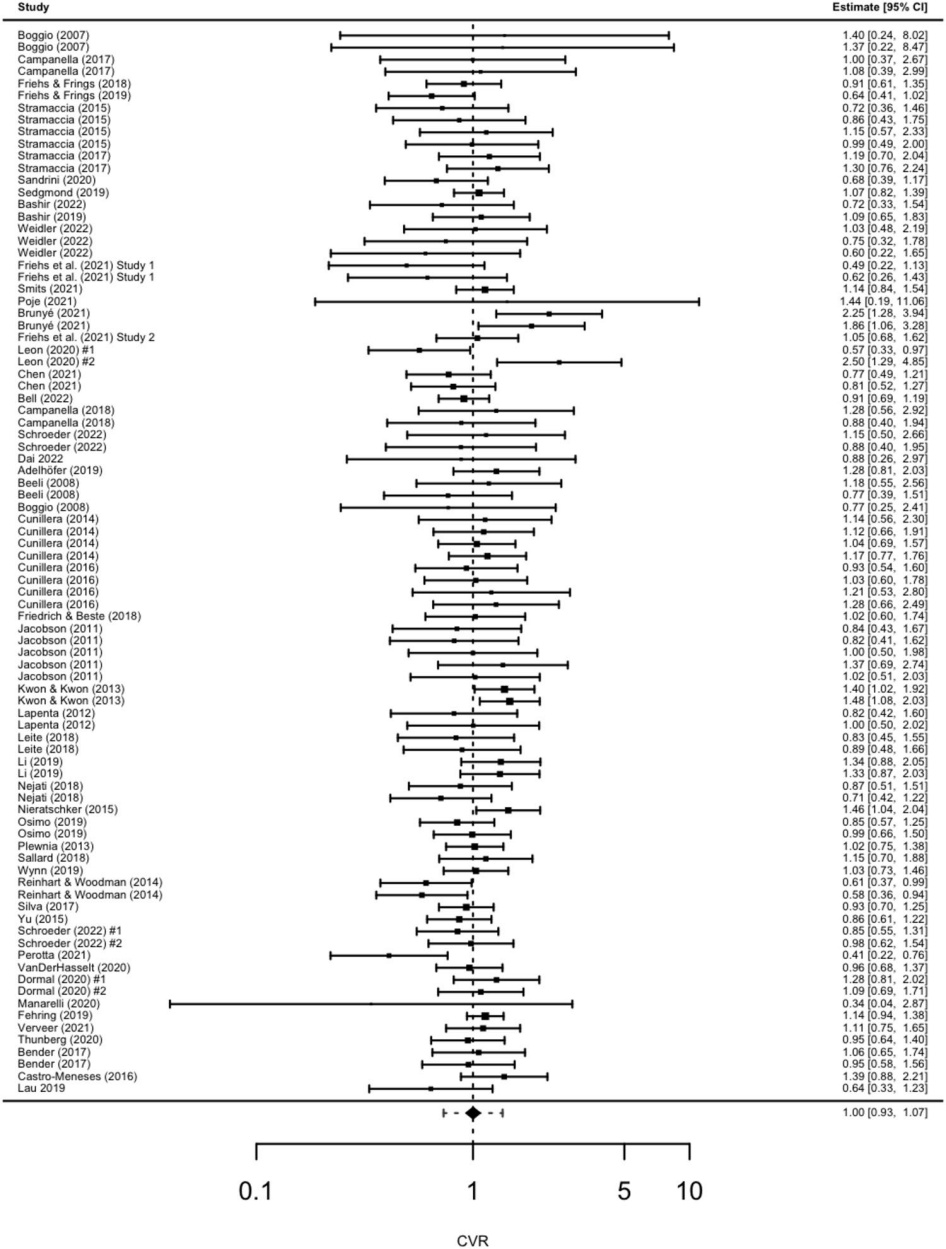
Forest plot of CVR multi-level meta-analysis.

The MLMA revealed that variance between active and sham tDCS did not differ (CVR = 1, p = 0.975, 95% CI [0.93, 1.07]) (see Table 1). All moderator tests were non-significant, which suggests that the hypothesis of equal CVR across comparisons cannot be rejected. Hence, CVR can be considered homogeneous across all studies or comparisons. The proportion of studies showing a CVR larger than 1 was 50%. Each comparison of CVR can be inspected in the forest plot (Fig. 1). The Q-test of heterogeneity, displayed in supplementary materials Table 6, was non-significant (QE = 96.65, *p* = 0.225).

**Table 1:** Multi-level meta-analyses of variance ratios.

Null model	CVR	*p*	AIC	BIC
	1	*0.975*	42.89	52.76

Moderator variable	QM	*p*	AIC	BIC
Task	0.01	0.929	45.28	57.56
Polarity	0.30	0.586	45.13	57.40
Target location	4.77	0.445	49.65	71.31
Return electrode	1.25	0.741	48.02	65.04
Timing	1.46	0.227	43.80	56.07
Current density	0.11	0.747	45.63	57.90
Duration	3.57	0.059	41.85	54.12
Blinding	0.44	0.933	50.94	67.95

Number of studies = 53, number of effects = 88, CVR = coefficient of variation ratio (lnCVR was exponentiated by *e*), QM = Moderator test, AIC = Akaikes information criterion, BIC = Bayesian information criterion.

### Sensitivity analyses

To validate the robustness of our findings, we conducted sensitivity analyses by using different ITCCs than 0.58. Using a correlation of 0.3 showed no difference in variance between active and sham tDCS (CVR = 1, *p* = 0.925, 95% CI [0.93, 1.08]). Moderator tests showed that duration of stimulation emerged as a significant moderator (CVR = 0.99, *p* = 0.038 95% CI [− 0.97, 1]). All other moderators were not significantly associated with the CVR (see Table 2). Similarly, using a correlation coefficient of 0.8 revealed no difference in variation between active and sham tDCS. In addition, no moderator showed a significant contribution (see Table 3). Cochranes risk of bias assessment identified seven studies with higher risk of bias (see in supplementary materials Table 7). Excluding these studies did not change the overall results (CVR = 0.98, *p* = 0.677, 95% CI [0.91, 1.06]).

**Table 2:** Sensitivity analysis with an intra-trial correlation coefficient of 0.3.

Null model	CVR	*p*	AIC	BIC
	1	*0.925*	50.11	59.98

Moderator variable	QM	*p*	AIC	BIC
Task	0.01	0.924	52.39	64.66
Polarity	0.43	0.510	52.06	64.33
Target location	5.10	0.404	56.47	78.13
Return electrode	1.48	0.688	54.79	71.81
Timing	1.39	0.239	51.02	63.30
Current density	0.09	0.768	52.70	64.98
Duration	4.29	0.038	48.50	60.78
Blinding	0.55	0.909	57.95	74.96

Number of studies = 53, number of effects = 88, CVR = coefficient of variation ratio (lnCVR was exponentiated), QM = Moderator test, AIC = Akikes information criterion, BIC = Bayesian information criterion, intra-trial correlation coefficient = 0.3.

**Table 3:** Sensitivity analysis with an intra-trial correlation coefficient of 0.8.

Null model	CVR	*p*	AIC	BIC
	1	*0.932*	28.96	33.89

Moderator variable	QM	*p*	AIC	BIC
Task	0.03	0.863	35.48	47.75
Polarity	0.14	0.708	35.65	47.93
Target location	4.26	0.513	40.20	61.86
Return electrode	0.94	0.816	38.59	55.61
Timing	1.52	0.217	33.86	46.13
Current density	0.15	0.696	35.81	48.08
Duration	2.61	0.107	32.73	45.00
Blinding	0.30	0.960	41.12	58.13

Number of studies = 53, number of effects = 88., CVR = coefficient of variation ratio (lnCVR was exponentiated), QM = Moderator test, AIC = Akikes information criterion, BIC = Bayesian information criterion, intra-trial correlation coefficient = 0.8.

### Hedges g

Calculating mean differences with hedges g showed no significant tDCS effect on response inhibition (g = − 0.11, p = 0.091, 95% CI [− 0.24, 0.018]) (see Table 4). Q-test of cross study heterogeneity provided evidence for the presence of increased heterogeneity (QE = 165.76, *p* < 0.0001). This effect suggests the null hypothesis that all true mean effect sizes (hedges g) are equal across studies or comparisons can be rejected. Hence, we expect that hedges g is heterogeneous across studies or comparisons.

**Table 4:** Sensitivity analysis with an intra-trial correlation coefficient of 0.8.

Null model	Hedges g	*p*	AIC	BIC
	− 0.11	*0.091*	132.82	142.68

Moderator variable	QM	*p*	AIC	BIC
Task	6.93	0.031*	130.43	142.70
Polarity	2.97	0.227	134.29	146.56
Target location	3.53	0.740	138.58	160.24
Return electrode	4.42	0.352	134.42	151.43
Timing	2.82	0.244	133.94	146.21
Current density	0.39	0.534	134.03	146.30
Duration	0.34	0.563	133.51	145.78
Blinding	1.14	0.768	136.30	153.32

Number of studies = 53, number of effects = 88. QM = Moderator test, AIC = Akaikes information criterion, BIC = Bayesian information criterion.

### Moderators

#### Task

**Figure 2:**
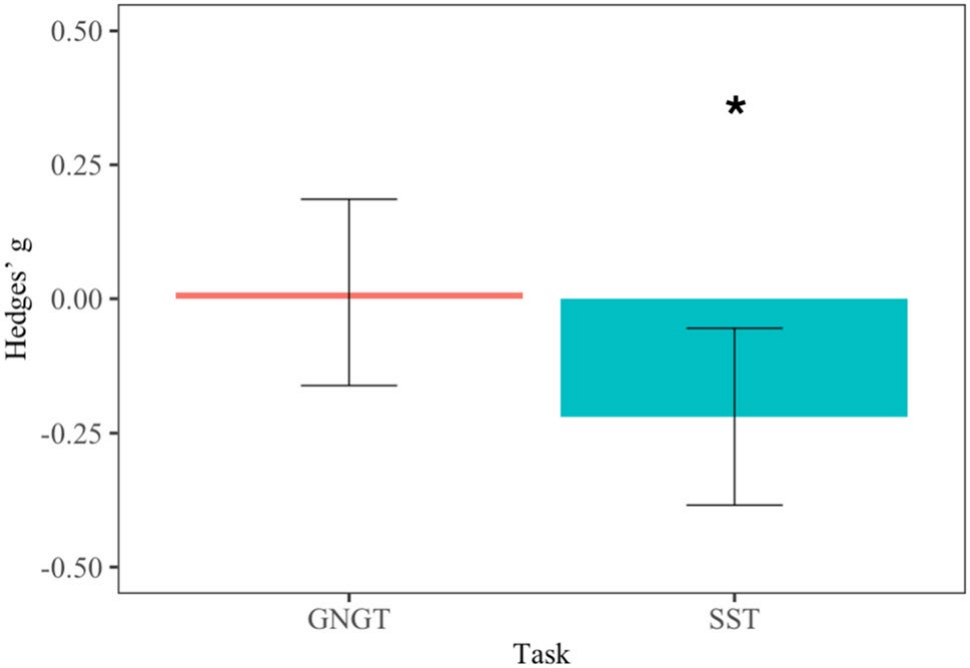
Bar graph displaying effect sizes of transcranial direct current stimulation effects on performance in the Stop Signal Task (SST) and Go/NoGo Task (GNGT). Performance in the GNGT did not significantly differ between active and sham tDCS (no difference from zero; p = 0.890), whereas performance in the SST was significantly improved in active as compared to sham tDCS (significant difference from zero; *p* = 0.01).

Task emerged as a significant moderator of tDCS effects on response inhibition. The standardized mean differences of comparisons between active and sham tDCS utilizing the SST showed a significant departure from zero (g = − 0.22, *p* = 0.01, 95% CI [− 0.38, − 0.06]). This indicates that the active tDCS condition was associated with a decrease in SSRT, which shows a small but significant improvement in response inhibition. In contrast, the standardized mean differences of comparisons using the GNGT did not show a significant difference from zero (g = 0.01, *p* = 0.890, 95% CI [− 0.16, 0.19]), indicating that FA did not differ between active and sham tDCS. This suggests that there was no improvement in response inhibition. Difference between tasks can be seen in Fig. 2. However, residual heterogeneity was increased (QE = 155.56, *p* < 0.0001). Testing the remaining moderators for significance showed no results (see Table 4). Q-tests of heterogeneity were all significant (see supplementary materials Table 8).

#### Publication bias

**Figure 3:**
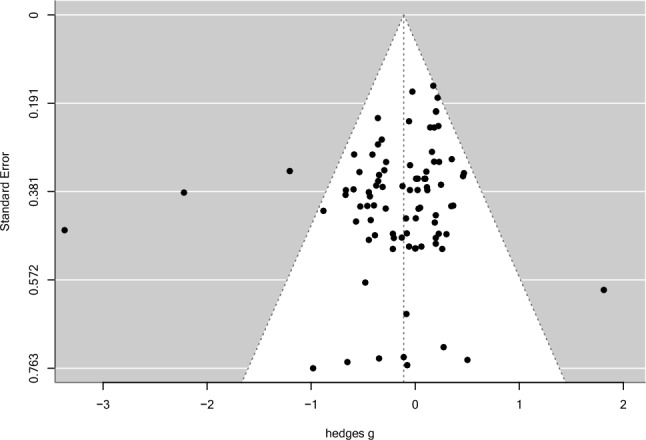
Funnel plot of effect sizes and standard error differences (hedges g).

Assessing publication bias revealed no severe bias. Visually inspecting funnel plots (see Fig. 3) and using Gregg’s test (*z* = − 1.12, *p* = 0.263) suggest that there is no evidence of funnel plot asymmetry, and thus no small sample bias. In addition, a rank correlation test showed no significance (τ = − 0.07, *p* = 0.371). Risk assessment for potential bias was conducted with the Cochrane risk assessment tool^40^. Cochrane risk of bias assessment identified seven studies that pose an overall higher risk of bias (see supplementary Table 7).

## Discussion

This meta-analysis aimed to compare variances in response inhibition performance between active and sham tDCS. Contrary to our hypothesis, no significant difference in variance between active and sham tDCS was found (CVR = 1). The number of studies showing higher variability in active tDCS (CVR > 1) and higher variability in sham tDCS (CVR < 1) were evenly distributed. None of the moderator variables were significant. Our secondary aim was to validate results from a previous meta-analysis^20^ focusing on mean effects. In contrast to Schroeder et al.^20^, no effects of tDCS on response inhibition were observed. However, and in line with^20^, task significantly moderated tDCS effects on response inhibition. Specifically, performance in the SST was significantly improved by active tDCS but not in the GNGT.

Active and sham tDCS groups did not differ in their variability in response inhibition, indicated by equal variances between active and sham tDCS. Specifically, 50 percent of the comparisons showed higher variances of active over sham tDCS whereas the other 50 percent showed the lower variance in active relative to sham tDCS. This pattern suggests that CVR was evenly distributed around 1, which could be attributable to chance or factors that we did not account for. None of the investigated moderators could explain any trend of this distribution. Therefore, variation in inter-individual responsiveness to tDCS does not appear to explain the heterogeneity in the literature regarding response inhibition.

The equal distribution of studies with a CVR of above and below one, plus the notably wide range of CVR [0.34; 2.5] suggest that studies may be underpowered and effects estimates are inaccurate. Nonetheless, there could be a number of factors other than individual differences that are associated with heterogeneous findings regarding tDCS efficacy. First, increased stimulation duration may be associated with a reversal of anodal or cathodal effects. In our data, stimulation duration was not found to be a significant moderator. It is worth noting, however, that earlier studies have demonstrated that applying anodal tDCS for over 20 min can have reverse effects and result in inhibition^41^. Similar findings were observed with cathodal tDCS, as a 20-min stimulation elicited excitatory effects^42^. This effect might contribute to a small amount of heterogeneity^43^, but it cannot be generalized to the majority of studies where tDCS delivers the intended effects. Circumstances under which stimulation duration can have reversed effects require further clarification.

Second, cathodal tDCS specifically has been shown to account for heterogenous findings in studies using cognitive tasks. Research investigating tDCS effects on motor and cognitive functions revealed that cathodal tDCS was associated with the expected inhibitory effect in studies investigating motor responses but not studies addressing cognitive-behavioral domains such as language, memory, and executive functions^14^. Furthermore, a meta-analysis showed that cathodal HD-tDCS effects on impulsivity and other cognitive functions were mixed and reported that heterogeneity within studies exceeded heterogeneity between studies^44^. This might suggest there are factors within studies, such as individual differences and not difference between studies, such as methodology differences that contribute to mixed findings. However, this is not in line with our results. Nonetheless, the effect of cathodal tDCS may be biased in our data, because it may be underrepresented due to the low number of studies using cathodal tDCS (21 comparison) compared to anodal tDCS (67 comparison).

Finally, the majority of included studies in our data recruited healthy individuals, where response inhibition is typically preserved. Individuals with intact response inhibition perform relatively well and may display less heterogeneous responses to tDCS. Patient groups may react differently since mental health conditions are variable and may introduce more variance^31,45,46^. On the other hand, a meta-analysis revealed that tDCS treatment effect variability in patients did not differ between active and sham tDCS in all patient groups. Only Schizophrenia was associated with a modest increase of variance in active versus sham tDCS^36^. Our data did not permit a subgroup analysis comparing patients to healthy participants due to the low number of patient samples. Qualitative inspections suggest that CVR may not show a systematic increase in patients.

### Standardized mean differences

Overall, there was no mean difference between active and sham tDCS. This finding is in contrast with previous research, which supported a small effect of tDCS on response inhibition^20^. This may be explained by a factor that cancelled out the effect. For instance, the moderator analysis revealed that tDCS effects on response inhibition are task dependent. For the SST, active tDCS was associated with improved performance compared to sham tDCS, while performance in the GNGT was comparable between active and sham tDCS. In line with previous research^20^, the effect is small but robust.

FMRI meta-analysis indicate that SST and GNGT are associated with brain activity in the rIFG^47^ and pre-supplementary motor areas^48^. Nonetheless, both tasks exhibit differences in signal processing, which may explain the difference in tDCS effects. Whereas the SST involves recruitment of frontal control components prior to stimulus detection, the GNGT might involve motor components at later stages^49^. Furthermore, differences in neural recruitment may stem from disparities in task demands. Both tasks usually present different types of stimuli, which may require distinct cognitive demands. Indeed, while the SST presents the go stimuli and immediate stop signal during stop trials, the GNGT replaces the go with the no-go stimulus^49^. Moreover, the SST usually requires participants to respond by indicating a direction, whereas the GNGT often involves letters or pictures. GNGT studies were shown to vary in task complexity, where increased complexity requires higher demands of working memory^50^ and, thus, additional cognitive resources. Most studies included in this meta-analysis stimulated prefrontal regions, which may be more beneficial for SST performance than for GNGT and could contribute to differences in tDCS effectiveness. Further research is needed to understand the mechanisms behind each task and how tDCS may affect them.

Our prediction that the position of the electrodes would moderate the relationship between tDCS effects and response inhibition was not supported. Unlike a previous meta-analysis^20^, target and reference electrodes were not identified as significant moderators. This lack of effect may be explained by the problem that clustering and simplifying variables could result in loss of information. Yet, using the precise location of target and reference electrodes would increase the number of levels within a variable, which may result in a power problem. The parameter space is, yet, too large to include each individual location. Decisions on using the optimal location for modulating response inhibition may also be informed by different neural mechanisms underlying SST and GNGT performance.

## Limitations

The findings of this meta-analysis must be considered under the following limitations. First, the majority of included studies suffers from small sample sizes, which reduces the precision of their respective effect sizes^51^. Second, bias assessment found low overall risk for two studies (~ 4%) and only 5 studies (~ 9%) were preregistered. Hence, transparency in the reporting of the reviewed tDCS literature was compromised, and, in combination with small sample sizes, particularly at risk for overestimated effect sizes^51^. We note that although a test for small study effects (publication bias) was not significant, this test itself is limited by the number of included studies (here 53) and hence a non-significant result does not exclude an overestimation in effect sizes^52^. Third, we imputed a correlation coefficient for intra-trial correlations. To calculate such a coefficient, we relied on paired sample t-tests, which were not conducted in all studies. We used a subset of six studies that provided a paired t-test, and averaged those correlations, which may be a source of inaccuracy in the calculation of this coefficient. Nonetheless, our ITCC of 0.58 was similar to coefficients from previous research (0.59) investigating variance in tDCS responses^36^. Fourth, we used single session tDCS studies, which do not include repeated measurements. Longitudinal designs with multiple sessions may be more beneficial as they can capture effects over time which should increase the robustness of the observed effects. Finally, tDCS parameters were heterogeneous. For instance, only three studies used HD-tDCS, which is not sufficient to draw reliable conclusions. This meta-analysis was not pre-registered.

## Implications and future directions

Our discussion highlights the importance of studying sources of heterogeneity regarding inter-individual. Factors that increase heterogeneity and potentially contribute to mixed findings need to be identified. Furthermore, research should allow for larger samples and, importantly, include more patient groups. Additionally, information on multiple sessions may become increasingly more available and meta-analysis focusing on multi-session tDCS may account for how tDCS effects last in the long run. Finally, methodology should be considered as the priority. Multicenter studies with clinical populations may encompass various tDCS montages for comparative analysis to determine important factors for successful tDCS administration. The easy and affordable use in clinical and forensic settings make tDCS a promising tool as an add-on-therapy to modulate response inhibition. A recently formed consortium may help promoting standardization and allow future mega-analyses on more harmonized data sets^53^. Lastly, to provide sufficient transparency and mitigate risks for biases, studies should be preregistered, and ideally peer-reviewed before data collection as so-called Registered Reports that provide an acceptance in principle independent of statistical outcomes^53,54^.

## Conclusion

We conclude that there is no difference in variances between active and sham tDCS conditions. This absence of a difference suggests that, for response inhibition, inter-individual variability may not account for heterogeneity of results in the literature. Uncertainty persists regarding factors, both between and within studies, that may contribute to heterogeneity in tDCS outcomes. Mean tDCS effects on response inhibition did not differ between active and sham conditions. Task emerged as a significant moderator, revealing that studies using the SST showed significantly improved response inhibition in the active tDCS condition. Studies using the GNGT, on the other hand, showed no differences between active and sham tDCS. Therefore, tDCS seems effective in improving response inhibition measured with the SST.

## Methods

### Eligibility criteria

This meta-analysis included single session tDCS studies that used the stop-signal task (SST) and/or the Go-NoGo task (GNGT). Studies that used parallel or crossover designs qualified for inclusion. Thus, eligible studies were single or double-blind and sham-controlled. Studies with only an active control condition were excluded. We included studies using healthy individuals and/or neurological and psychiatric patients.

### Search strategy

Methodological steps adhered to the Preferred Reporting Items for Systematic reviews (PRISMA)^55^. The literature search in PubMed and Web of Science was conducted ended on the 21.04.2023. Review and meta-analyses were examined for further studies but discarded for the meta-analysis. Three articles^56–58^ were found in another meta-analysis^20^. Search terms used in PubMed and Web of Science databases are displayed in Table 5.

**Table 5:** Terms for the PRISMA literature search.

Search step	PubMed (abstract/title)	Web of science (abstract)
1	“transcranial direct current stimulation” OR “trans-cranial direct current stimulation” OR “tDCS” OR “brain stimulation”	“transcranial direct current stimulation” OR “trans-cranial direct current stimulation” OR “tDCS” OR “brain stimulation”
	AND	AND
2	“response inhibition” OR “action inhibition” OR inhibitory control” OR “inhibitory response control” OR “inhibitory action control” OR “impulse control” OR “self-control” OR “motor inhibition” OR “inhibitory motor control” OR “impuls*”	“response inhibition” OR “action inhibition” OR inhibitory control” OR “inhibitory response control” OR “inhibitory action control” OR “impulse control” OR “self-control” OR “motor inhibition” OR “inhibitory motor control” OR “impuls*”
	OR	OR
3	“stop-signal-task" OR “SST” OR “stop-task” OR “stop signal reaction time” OR “SSRT”	“stop-signal-task" OR “SST” OR “stop-task” OR “stop signal reaction time” OR “SSRT”
	OR	OR
4	“go-nogo” OR “go-/nogo” OR “go/no-go” OR “go-no-go" OR “GNG” OR “GNGT”	“go-nogo” OR “go-/nogo” OR “go/no-go” OR “go-no-go" OR “GNG” OR “GNGT”
5	Apply inclusion criteria (applied in the following order): a. Original research article (i.e. no review, metal-analysis) b. English language c. Human subjects d. Stop Signal and/or Go No-Go task e. tDCS study	

### Study selection

**Figure 4:**
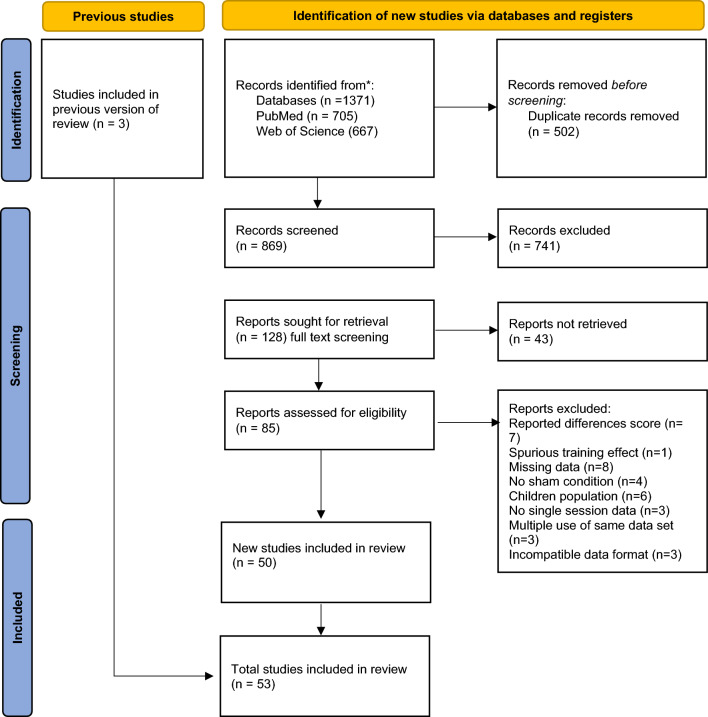
PRISMA flowchart of the literature search.

Studies were selected based on the eligibility criteria described above. After the exclusion of duplicates, all non-eligible articles were discarded via title and abstract screening—and subsequently full text screening (Fig. 4). Two raters—LL and CG—conducted the literature screening independently from each other. Inter-rater reliability was *κ* = 0.848. Rater DM screened the remaining articles that did not meet initial agreement. An overview of all included studies can be found in the supplementary material (Supplementary Table 9).

### TDCS parameters

The extraction of tDCS parameters was based on previous research. We selected polarity consisting of anodal and cathodal stimulation^14^, as well as target and return electrode position, study design, timing, current density, intensity, stimulation duration, blinding, and task^20^.

### Outcome variables

The SST and GNGT were selected as the primary behavioral measure for assessing response inhibition. The studies of interest included stop-signal reaction time (SSRT) and false alarms (FA) for the SST and GNGT, respectively. SSRT is a measure that is usually reported in milliseconds as it was used in this analysis. FA are commonly reported as the number or percentage of false alarms.

### Data extraction

Summary data of both behavioral measures have been used to extract mean (M), standard deviation (SD) and sample size (N) for active and sham tDCS conditions. If data were not found, we searched through other meta-analyses. One meta-analysis^20^ provides a comprehensive data summary for response inhibition. If summary data were only available in the form of figures, we used the Webplotdigitizer^59^ to extract M and SD. Standard errors (SEs) and 95% confidence intervals were transformed into SDs (see supplementary Formulas 1 and 2). Summary statistics limited to pre-post differences had to be excluded due to their incompatibility with lnCVR. The final dataset included 53 studies and 88 comparisons. The final data set and the R-script are available in the supplementary materials.

### Data pre-processing

Some studies provided more than one comparison, comparing multiple active conditions to one sham group. To account for the multiple use of one sham group we divided N of the sham group by the number of trial arms^36,60^. Subsequently, to calculate the variance ratio for crossover studies, we used an intra-trial correlation coefficient (ITCC, see supplementary Formula 3). This ITCC considers intra-trial correlation to account for the relationship between active and sham sessions underwent by each participant^61^. Several studies did not report the necessary SD of mean difference of paired t-tests between active and sham groups, which is crucial for calculating the ITCC (see supplementary materials Formula 4). The ITCC was calculated based on 8 comparisons deemed suitable for this imputation. The average coefficient was 0.58, which was used to account for the relationship between active and sham tDCS.

### Effect sizes for variances

The calculation of effect sizes was based on previous research using the lnCVR^35,38,62^. The central idea of lnCVR is to divide the SD of the active group by the SD of the sham group to gain information on which variance is larger. One of its major advantages is that it accounts for mean–variance relationships^35,62^. Senior et al.^62^ propose different effect size calculations for independent—or parallel—designs, and dependent—or crossover—designs. The formulas for the effect size and sampling distribution in this meta-analysis were validated by previous research^62^. All formulas are presented in the supplementary materials (Formulas 5, 6, 7 and 8 respectively).

### Multilevel meta-analysis for lnCVR

We conducted a multi-level meta-analysis (MLMA) with the metafor package^63^ implemented in R Version 2023.12 that can account for dependent effect sizes coming from the same study. The effect sizes and sampling distribution based on the lnCVR were entered in a MLMA. The restricted maximum likelihood method was used for model fitting. This mixed-effects model included three random factors: study ID, study design, and publication year. As fixed factors the following moderators were included: tDCS polarity (anodal vs cathodal), timing (online vs offline), task (SST vs GNGT), current density, return electrode location and stimulation location. Moderators were individually added to the MLMA, meaning one model was calculated for each moderator. Significant contribution of each moderator was evaluated on the basis of their *p*-values^20,44^. Q-tests of heterogeneity were inspected.

### Multilevel meta-analysis of standardized mean differences

Another MLMA was conducted to investigate standardized mean effects of tDCS on response inhibition and followed the same steps as the previous MLMA. The dependent variable was hedges g, which measured differences in mean task performance between active and sham tDCS. Heterogeneity of mean effect was assessed with Cochranes Q-test. Data and code are available in the supplementary materials.

### Supplementary Information

Supplementary Information 1.

Supplementary Information 2.

Supplementary Information 3.

Supplementary Information 4.

Supplementary Information 5.

## Data Availability

Data and the analysis script are provided within the supplementary materials.
