# Supplementary material for: Meta-analysis of variance in tDCS effects on response inhibition
Source: Sci Rep. 2024 Aug 19;14:19197. doi: 10.1038/s41598-024-70065-7 (PMC11333595; doi:10.1038/s41598-024-70065-7)
Supplement: Supplementary file 2 — Supplementary Information 2. [file 41598_2024_70065_MOESM2_ESM.docx]

The final data set consisted of 53 studies ^[1–53]^. Furthermore, some studies identified to meet the initial inclusion criteria were excluded based on the following reason: outcome estimate was based on only difference scores between pre and post tDCS session ^[54–60]^, non, incomplete, or unclear information on the outcome variable data ^[61–68]^, no sham condition ^[69–72]^, only multi-session data ^[73–75]^, incompatible data ^[76–78]^, appearance of using the same data set multiple times ^[79–81]^, children’s population ^[82–87]^ and a spurious training effect ^[88]^.

**References**

1. Adelhöfer, N., Mückschel, M., Teufert, B., Ziemssen, T. & Beste, C. Anodal tDCS affects neuromodulatory effects of the norepinephrine system on superior frontal theta activity during response inhibition. *Brain Struct. Funct.* **224**, 1291–1300 (2019).

2. Bashir, S. *et al.* Anodal Transcranial Direct Current Stimulation (tDCS) Over the Primary Motor Cortex (M1) Enhances Motor Response Inhibition and Visual Recognition Memory. *Med. Sci. Monit. Basic Res.* **28**, e934180 (2022).

3. Bashir, S. *et al.* Cognitive function assessment during 2 mA transcranial direct current stimulation in DLPFC in healthy volunteers. *Physiol. Rep.* **7**, e14264 (2019).

4. Beeli, G., Casutt, G., Baumgartner, T. & Jancke, L. Modulating presence and impulsiveness by external stimulation of the brain. *Behav. BRAIN Funct.* **4**, (2008).

5. Bell, S., Turner, B., Sawaki, L. & DeWall, N. When brain stimulation backfires: the effects of prefrontal cortex stimulation on impulsivity. *Soc. Cogn. Affect. Neurosci.* **17**, 101–108 (2022).

6. Bender, A., Filmer, H. & Dux, P. Transcranial direct current stimulation of superior medial frontal cortex disrupts response selection during proactive response inhibition. *NEUROIMAGE* **158**, 455–465 (2017).

7. Boggio, P. S., Rocha, R. R., da Silva, M. T. & Fregni, F. Differential modulatory effects of transcranial direct current stimulation on a facial expression go-no-go task in males and females. *Neurosci. Lett.* **447**, 101–105 (2008).

8. Boggio, P. S. *et al.* Go-no-go task performance improvement after anodal transcranial DC stimulation of the left dorsolateral prefrontal cortex in major depression. *J. Affect. Disord.* **101**, 91–98 (2007).

9. Brunye, T., Elliott, G. & Loverro, K. Examining state-dependent effects of transcranial direct current stimulation on visual search and executive function tasks. *NEUROREPORT* **32**, 1–7 (2021).

10. Campanella, S. *et al.* Short-Term Impact of tDCS Over the Right Inferior Frontal Cortex on Impulsive Responses in a Go/No-go Task. *Clin. EEG Neurosci.* **49**, 398–406 (2018).

11. Campanella, S. *et al.* Transcranial Direct Current Stimulation Over the Right Frontal Inferior Cortex Decreases Neural Activity Needed to Achieve Inhibition: A Double-Blind ERP Study in a Male Population. *Clin. EEG Neurosci.* **48**, 176–188 (2017).

12. Castro-Meneses, L., Johnson, B. & Sowman, P. Vocal response inhibition is enhanced by anodal tDCS over the right prefrontal cortex. *Exp. BRAIN Res.* **234**, 185–195 (2016).

13. Chen, T. *et al.* Transcranial direct current stimulation of the right dorsolateral prefrontal cortex improves response inhibition. *Int. J. Psychophysiol.* **162**, 34–39 (2021).

14. Cunillera, T., Brignani, D., Cucurell, D., Fuentemilla, L. & Miniussi, C. The right inferior frontal cortex in response inhibition: A tDCS-ERP co-registration study. *NeuroImage* **140**, 66–75 (2016).

15. Cunillera, T., Fuentemilla, L., Brignani, D., Cucurell, D. & Miniussi, C. A simultaneous modulation of reactive and proactive inhibition processes by anodal tDCS on the right inferior frontal cortex. *PloS One* **9**, e113537 (2014).

16. Dai, J. *et al.* The neuroelectrophysiological and behavioral effects of transcranial direct current stimulation on executive vigilance under a continuous monotonous condition. *Front. Neurosci.* **16**, 910457 (2022).

17. Dormal, V., Lannoy, S., Bollen, Z., D’Hondt, F. & Maurage, P. Can we boost attention and inhibition in binge drinking? Electrophysiological impact of neurocognitive stimulation. *Psychopharmacology (Berl.)* **237**, 1493–1505 (2020).

18. Fehring, D. *et al.* Interaction of task-related learning and transcranial direct current stimulation of the prefrontal cortex in modulating executive functions. *NEUROPSYCHOLOGIA* **131**, 148–159 (2019).

19. Friedrich, J. & Beste, C. Paradoxical, causal effects of sensory gain modulation on motor inhibitory control - a tDCS, EEG-source localization study. *Sci. Rep.* **8**, 17486 (2018).

20. Friehs, M. A., Dechant, M., Vedress, S., Frings, C. & Mandryk, R. Shocking advantage! Improving digital game performance using non-invasive brain stimulation. *Int. J. Hum.-Comput. Stud.* **148**, (2021).

21. Friehs, M. A., Brauner, L. & Frings, C. Dual-tDCS over the right prefrontal cortex does not modulate stop-signal task performance. *Exp. Brain Res.* **239**, 811–820 (2021).

22. Friehs, M. A. & Frings, C. Cathodal tDCS increases stop-signal reaction time. *Cogn. Affect. Behav. Neurosci.* **19**, 1129–1142 (2019).

23. Friehs, M. A. & Frings, C. Pimping inhibition: Anodal tDCS enhances stop-signal reaction time. *J. Exp. Psychol. Hum. Percept. Perform.* **44**, 1933–1945 (2018).

24. Jacobson, L., Koslowsky, M. & Lavidor, M. tDCS polarity effects in motor and cognitive domains: a meta-analytical review. *Exp. Brain Res.* **216**, 1–10 (2012).

25. Kwon, Y. H. & Kwon, J. W. Response Inhibition Induced in the Stop-signal Task by Transcranial Direct Current Stimulation of the Pre-supplementary Motor Area and Primary Sensoriomotor Cortex. *J. Phys. Ther. Sci.* **25**, 1083–1086 (2013).

26. Lapenta, O. M., Fregni, F., Oberman, L. M. & Boggio, P. S. Bilateral temporal cortex transcranial direct current stimulation worsens male performance in a multisensory integration task. *Neurosci. Lett.* **527**, 105–109 (2012).

27. Lau, C.-I. *et al.* Effect of single-session transcranial direct current stimulation on cognition in Parkinson’s disease. *CNS Neurosci. Ther.* **25**, 1237–1243 (2019).

28. Leite, J. *et al.* The differential effects of unihemispheric and bihemispheric tDCS over the inferior frontal gyrus on proactive control. *Neurosci. Res.* **130**, 39–46 (2018).

29. Leon, J. *et al.* Transcranial direct current stimulation improves risky decision making in women but not in men: A sham-controlled study. *Behav. BRAIN Res.* **382**, (2020).

30. Li, L. M. *et al.* Cognitive enhancement with Salience Network electrical stimulation is influenced by network structural connectivity. *NeuroImage* **185**, 425–433 (2019).

31. Mannarelli, D. *et al.* Effects of Cerebellar tDCS on Inhibitory Control: Evidence from a Go/NoGo Task. *Cerebellum Lond. Engl.* **19**, 788–798 (2020).

32. Nejati, V., Salehinejad, M. & Nitsche, M. Interaction of the Left Dorsolateral Prefrontal Cortex (l-DLPFC) and Right Orbitofrontal Cortex (OFC) in Hot and Cold Executive Functions: Evidence from Transcranial Direct Current Stimulation (tDCS). *NEUROSCIENCE* **369**, 109–123 (2018).

33. Nieratschker, V., Kiefer, C., Giel, K., Krüger, R. & Plewnia, C. The COMT Val/Met polymorphism modulates effects of tDCS on response inhibition. *Brain Stimulat.* **8**, 283–288 (2015).

34. Osimo, S. A., Korb, S. & Aiello, M. Obesity, subliminal perception and inhibition: Neuromodulation of the prefrontal cortex. *Behav. Res. Ther.* **119**, 103408 (2019).

35. Perrotta, D., Bianco, V., Berchicci, M., Quinzi, F. & Perri, R. L. Anodal tDCS over the dorsolateral prefrontal cortex reduces Stroop errors. A comparison of different tasks and designs. *Behav. Brain Res.* **405**, 113215 (2021).

36. Plewnia, C. *et al.* Effects of transcranial direct current stimulation (tDCS) on executive functions: Influence of COMT Val/Met polymorphism. *CORTEX* **49**, 1801–1807 (2013).

37. Poje, A. *et al.* Effects of Transcranial Direct Current Stimulation (tDCS) on Go/NoGo Performance Using Food and Non-Food Stimuli in Patients with Prader-Willi Syndrome. *BRAIN Sci.* **11**, (2021).

38. Reinhart, R. M. & Woodman, G. F. Causal control of medial–frontal cortex governs electrophysiological and behavioral indices of performance monitoring and learning. *J. Neurosci.* **34**, 4214–4227 (2014).

39. Sallard, E., Mouthon, M., De Pretto, M. & Spierer, L. Modulation of inhibitory control by prefrontal anodal tDCS: A crossover double-blind sham-controlled fMRI study. *PLOS ONE* **13**, (2018).

40. Sandrini, M. *et al.* Transcranial direct current stimulation facilitates response inhibition through dynamic modulation of the fronto-basal ganglia network. *Brain Stimulat.* **13**, 96–104 (2020).

41. Schroeder, P. A., Farshad, M. & Svaldi, J. Anodal stimulation of inhibitory control and craving in satiated restrained eaters. *Nutr. Neurosci.* **26**, 403–413 (2023).

42. Schroeder, P. A., Seewald, A. & Svaldi, J. Spotlight on the Left Frontal Cortex: No Evidence for Response Inhibition from Cathodal High-Definition transcranial Direct Current Stimulation over Left Inferior Frontal Gyrus or Left Dorsolateral Prefrontal Cortex. *J. Cogn. Neurosci.* **34**, 1090–1102 (2022).

43. Sedgmond, J. *et al.* Prefrontal brain stimulation during food-related inhibition training: effects on food craving, food consumption and inhibitory control. *R. Soc. Open Sci.* **6**, 181186 (2019).

44. Silva, A. F. *et al.* Anodal transcranial direct current stimulation over the left dorsolateral prefrontal cortex modulates attention and pain in fibromyalgia: randomized clinical trial. *Sci. Rep.* **7**, 135 (2017).

45. Smits, F., de Kort, G. & Geuze, E. Acceptability of tDCS in treating stress-related mental health disorders: a mixed methods study among military patients and caregivers. *BMC PSYCHIATRY* **21**, (2021).

46. Stramaccia, D. F., Penolazzi, B., Altoè, G. & Galfano, G. TDCS over the right inferior frontal gyrus disrupts control of interference in memory: A retrieval-induced forgetting study. *Neurobiol. Learn. Mem.* **144**, 114–130 (2017).

47. Stramaccia, D. F. *et al.* Assessing the effects of tDCS over a delayed response inhibition task by targeting the right inferior frontal gyrus and right dorsolateral prefrontal cortex. *Exp. Brain Res.* **233**, 2283–2290 (2015).

48. Thunberg, C., Messel, M. S., Raud, L. & Huster, R. J. tDCS over the inferior frontal gyri and visual cortices did not improve response inhibition. *Sci. Rep.* **10**, 7749 (2020).

49. Vanderhasselt, M.-A. *et al.* Bifrontal tDCS applied to the dorsolateral prefrontal cortex in heavy drinkers: Influence on reward-triggered approach bias and alcohol consumption. *Brain Cogn.* **138**, 105512 (2020).

50. Verveer, I. *et al.* Modulation of control: Can HD-tDCS targeting the dACC reduce impulsivity? *BRAIN Res.* **1756**, (2021).

51. Weidler, C. *et al.* Consequences of prefrontal tDCS on inhibitory control and reactive aggression. *Soc. Cogn. Affect. Neurosci.* **17**, 120–130 (2022).

52. Wynn, S. C., Driessen, J. M. A., Glennon, J. C., Brazil, I. A. & Schutter, D. J. L. G. Cerebellar Transcranial Direct Current Stimulation Improves Reactive Response Inhibition in Healthy Volunteers. *Cerebellum Lond. Engl.* **18**, 983–988 (2019).

53. Yu, J., Tseng, P., Hung, D. L., Wu, S.-W. & Juan, C.-H. Brain stimulation improves cognitive control by modulating medial-frontal activity and preSMA-vmPFC functional connectivity. *Hum. Brain Mapp.* **36**, 4004–4015 (2015).

54. Cai, Y. *et al.* The Role of the Frontal and Parietal Cortex in Proactive and Reactive Inhibitory Control: A Transcranial Direct Current Stimulation Study. *J. Cogn. Neurosci.* **28**, 177–186 (2016).

55. Cosmo, C. *et al.* A Randomized, Double-Blind, Sham-Controlled Trial of Transcranial Direct Current Stimulation in Attention-Deficit/Hyperactivity Disorder. *PloS One* **10**, e0135371 (2015).

56. Dambacher, F. *et al.* No Effects of Bilateral tDCS over Inferior Frontal Gyrus on Response Inhibition and Aggression. *PloS One* **10**, e0132170 (2015).

57. Hogeveen, J. *et al.* Effects of High-Definition and Conventional tDCS on Response Inhibition. *Brain Stimulat.* **9**, 720–729 (2016).

58. Kang, E., Baek, M., Kim, S. & Paik, N. Non-invasive cortical stimulation improves post-stroke attention decline. *Restor. Neurol. Neurosci.* **27**, 645–650 (2009).

59. Mansouri, F. *et al.* Interactive effects of music and prefrontal cortex stimulation in modulating response inhibition. *Sci. Rep.* **7**, (2017).

60. Molero-Chamizo, A. *et al.* Poststimulation time interval-dependent effects of motor cortex anodal tDCS on reaction-time task performance. *Cogn. Affect. Behav. Neurosci.* **18**, 167–175 (2018).

61. Cecere, R., Bertini, C. & Làdavas, E. Differential contribution of cortical and subcortical visual pathways to the implicit processing of emotional faces: a tDCS study. *J. Neurosci. Off. J. Soc. Neurosci.* **33**, 6469–6475 (2013).

62. Chen, S., Jackson, T., Dong, D., Zhang, X. & Chen, H. Exploring effects of single-session anodal tDCS over the inferior frontal gyrus on responses to food cues and food cravings among highly disinhibited restrained eaters: A preliminary study. *Neurosci. Lett.* **706**, 211–216 (2019).

63. Conley, A. C. *et al.* Anodal tDCS over the Motor Cortex on Prepared and Unprepared Responses in Young Adults. *PloS One* **10**, e0124509 (2015).

64. Dubreuil-Vall, L. *et al.* Transcranial Direct Current Stimulation to the Left Dorsolateral Prefrontal Cortex Improves Cognitive Control in Patients With Attention- Deficit/Hyperactivity Disorder: A Randomized Behavioral and Neurophysiological Study. *Biol. PSYCHIATRY-Cogn. Neurosci. NEUROIMAGING* **6**, 439–448 (2021).

65. Guo, Z. *et al.* Multitarget high-definition transcranial direct current stimulation improves response inhibition more than single-target high-definition transcranial direct current stimulation in healthy participants. *Front. Neurosci.* **16**, (2022).

66. Khalil, R., Karim, A., Kondinska, A. & Godde, B. Effects of transcranial direct current stimulation of left and right inferior frontal gyrus on creative divergent thinking are moderated by changes in inhibition control. *BRAIN Struct. Funct.* **225**, 1691–1704 (2020).

67. Lapenta, O. M., Sierve, K. D., de Macedo, E. C., Fregni, F. & Boggio, P. S. Transcranial direct current stimulation modulates ERP-indexed inhibitory control and reduces food consumption. *Appetite* **83**, 42–48 (2014).

68. Ljubisavljevic, M., Basha, J. & Ismail, F. Y. The effects of prefrontal vs. parietal cortex transcranial direct current stimulation on craving, inhibition, and measures of self-esteem. *Front. Neurosci.* **16**, 998875 (2022).

69. Ditye, T., Jacobson, L., Walsh, V. & Lavidor, M. Modulating behavioral inhibition by tDCS combined with cognitive training. *Exp. BRAIN Res.* **219**, 363–368 (2012).

70. Hsu, T. *et al.* Modulating inhibitory control with direct current stimulation of the superior medial frontal cortex. *NEUROIMAGE* **56**, 2249–2257 (2011).

71. Liang, W.-K. *et al.* Revealing the brain’s adaptability and the transcranial direct current stimulation facilitating effect in inhibitory control by multiscale entropy. *NeuroImage* **90**, 218–234 (2014).

72. Sikström, S. *et al.* Self-Rated Attentiveness Interacts with Transcranial Direct Current Stimulation and Noise Stimulation in Reaction Time in a Go/No-Go Task. *Neural Plast.* **2016**, 5302538 (2016).

73. Dubuson, M. *et al.* Transcranial direct current stimulation combined with alcohol cue inhibitory control training reduces the risk of early alcohol relapse: A randomized-controlled clinical trial. *Brain Stimulat.* **14**, 1531–1543 (2021).

74. Shiga, K., Miyaguchi, S., Inukai, Y., Otsuru, N. & Onishi, H. Transcranial direct current stimulation over the right intraparietal sulcus improves response inhibition. *Behav. BRAIN Res.* **437**, (2023).

75. Verveer, I., van der Veen, F., Shahbabaie, A., Remmerswaal, D. & Franken, I. Multi-session electrical neuromodulation effects on craving, relapse and cognitive functions in cocaine use disorder: A randomized, sham-controlled tDCS study. *DRUG ALCOHOL Depend.* **217**, (2020).

76. Fujiyama, H., Tan, J., Puri, R. & Hinder, M. Influence of tDCS over right inferior frontal gyrus and pre-supplementary motor area on perceptual decision-making and response inhibition: A healthy ageing perspective. *Neurobiol. AGING* **109**, 11–21 (2022).

77. Müller, T., Shevchenko, Y., Gerhardt, S., Kiefer, F. & Vollstädt-Klein, S. The influence of perceived stress and self-control on efficacy of repeated transcranial direct current stimulation in non-treatment-seeking smokers. *Drug Alcohol Depend.* **226**, 108861 (2021).

78. Wu, D. *et al.* Initial performance modulates the effects of cathodal transcranial direct current stimulation (tDCS) over the right dorsolateral prefrontal cortex on inhibitory control. *BRAIN Res.* **1774**, (2022).

79. Fehring, D. J. *et al.* Investigating the sex-dependent effects of prefrontal cortex stimulation on response execution and inhibition. *Biol. Sex Differ.* **12**, 47 (2021).

80. Kwon, J. W. *et al.* The effect of transcranial direct current stimulation on the motor suppression in stop-signal task. *NeuroRehabilitation* **32**, 191–196 (2013).

81. Kwon, Y. & Kwon, J. Is transcranial direct current stimulation a potential method for improving response inhibition? *NEURAL Regen. Res.* **8**, 1048–1054 (2013).

82. Nejati, V. *et al.* Transcranial direct current stimulation (tDCS) alters the pattern of information processing in children with ADHD: Evidence from drift diffusion modeling. *Neurophysiol. Clin.-Clin. Neurophysiol.* **52**, 17–27 (2022).

83. Nejati, V., Movahed Alavi, M. & Nitsche, M. A. The Impact of Attention Deficit-hyperactivity Disorder Symptom Severity on the Effectiveness of Transcranial Direct Current Stimulation (tDCS) on Inhibitory Control. *Neuroscience* **466**, 248–257 (2021).

84. Nejati, V., Salehinejad, M. A., Nitsche, M. A., Najian, A. & Javadi, A.-H. Transcranial Direct Current Stimulation Improves Executive Dysfunctions in ADHD: Implications for Inhibitory Control, Interference Control, Working Memory, and Cognitive Flexibility. *J. Atten. Disord.* **24**, 1928–1943 (2020).

85. Salehinejad, M. A., Vosough, Y. & Nejati, V. The Impact of Bilateral Anodal tDCS over Left and Right DLPFC on Executive Functions in Children with ADHD. *Brain Sci.* **12**, (2022).

86. Salehinejad, M. A., Ghayerin, E., Nejati, V., Yavari, F. & Nitsche, M. A. Domain-specific Involvement of the Right Posterior Parietal Cortex in Attention Network and Attentional Control of ADHD: A Randomized, Cross-over, Sham-controlled tDCS Study. *Neuroscience* **444**, 149–159 (2020).

87. Soltaninejad, Z., Nejati, V. & Ekhtiari, H. Effect of Anodal and Cathodal Transcranial Direct Current Stimulation on DLPFC on Modulation of Inhibitory Control in ADHD. *J. Atten. Disord.* **23**, 325–332 (2019).

88. Filmer, H. L., Lyons, M., Mattingley, J. B. & Dux, P. E. Anodal tDCS applied during multitasking training leads to transferable performance gains. *Sci. Rep.* **7**, 12988 (2017).
