# Supplementary material for: Meta-analysis of variance in tDCS effects on response inhibition
Source: Sci Rep. 2024 Aug 19;14:19197. doi: 10.1038/s41598-024-70065-7 (PMC11333595; doi:10.1038/s41598-024-70065-7)
Supplement: Supplementary file 5 — Supplementary Information 5. [file 41598_2024_70065_MOESM5_ESM.docx]

**Supplementary materials**

**Formulas**

Formula 1. Standard error into standard deviation

$$SD=SE* \sqrt{N}$$

Formula 2. Confidence interval into standard deviation

$$SD = \frac{\sqrt{N}* (upper limit-lower limit)}{3.92}$$

Formula 3. Intra-trial correlation coefficient (ITCC)

$$ITCC=\frac{{{SD}_{active}}^{2}+ {{SD}_{sham}}^{2}- {{SD}_{\Delta}}^{2}}{2* {SD}_{active}* {SD}_{sham}}$$

Formula 4. Standard deviation of the difference between active and sham tDCS group

$${SD}_{\Delta}=\frac{M_{active}- M_{sham}}{t/\sqrt{N}}$$

Note. This formula is based on the conducted t-test.

Formula 5. Logarithm of the coefficient of variation ratio for studies with a parallel design

$$lnCVR=log(\frac{\frac{{SD}_{active}}{M_{active}}}{\frac{{SD}_{sham}}{M_{sham}}})+\left( \frac{1}{\left( 2*N_{active}-1 \right)- \left( 2*N_{sham}-1 \right)} \right)+0.5*(\frac{{{SD}_{sham}}^{2}}{N_{sham}* {M_{sham}}^{2}}-\frac{{{SD}_{active}}^{2}}{N_{active}* {M_{active}}^{2}}$$

Formula 6. Sampling distribution for studies with a parallel design

$$s^{2}=\frac{{{SD}_{sham}}^{2}}{N_{sham}*{M_{sham}}^{2}}+\frac{{{SD}_{sham}}^{4}}{2* {N_{sham}}^{2}* {M_{sham}}^{4}}+\frac{1}{2* {(N}_{sham}-1)}+\frac{1}{2* {{(N}_{sham}-1)}^{2}}+ \frac{{{SD}_{active}}^{2}}{N_{active}*{M_{active}}^{2}}+\frac{{{SD}_{active}}^{4}}{2* {N_{active}}^{2}* {M_{active}}^{4}}+\frac{1}{2* {(N}_{active}-1)}+\frac{1}{2* {{(N}_{sham}-1)}^{2}}$$

Formula 7. Logarithm of the coefficient of variation ratio for studies with a crossover design

$lnCVR= \log\left( \frac{\frac{{SD}_{active}}{M_{active}}}{\frac{{SD}_{sham}}{M_{sham}}} \right)- 0.5*(\frac{{{SD}_{sham}}^{2}}{N_{sham}* {M_{sham}}^{2}}-\frac{{{SD}_{active}}^{2}}{N_{active}* {M_{active}}^{2}})$

Formula 8. Sampling distribution for studies with a crossover design

$$s^{2}= \frac{{{SD}_{sham}}^{2}}{N_{sham}*{M_{sham}}^{2}}+ \frac{{{SD}_{active}}^{2}}{N_{active}*{M_{active}}^{2}}-ITCC* \frac{2*{SD}_{sham}* M_{sham}}{n* M_{sham}* M_{active}}+ \frac{1}{\left. n-1 \right.}-{ITCC}^{2}* \frac{1}{n-1}$$

Table 6.

Heterogeneity test CVR

| Moderator Variable |  |  |
| --- | --- | --- |
|  | QE | *p* |
| Null | 96.6450 | *0.2249* |
| Task | 96.5600 | 0.2048 |
| Polarity | 95.7059 | 0.2223 |
| Target Location | 88.8502 | 0.2835 |
| Return Electrode | 93.3156 | 0.2282 |
| Timing | 94.9484 | 0.2387 |
| Current Density | 96.5297 | 0.2054 |
| Duration | 88.4794 | 0.4059 |
| Blinding | 95.2594 | 0.1885 |
| Note. Number of studies = 53, number of effects = 88. QE = Test for Residual Heterogeneity | | |

Table 7

Cochrane risk of Bias assessment

| **Intention-to-treat** | **Study ID** | **D1** | **DS** | **D2** | **D3** | **D4** | **D5** | **Overall** |  |  |  |  |  |
| --- | --- | --- | --- | --- | --- | --- | --- | --- | --- | --- | --- | --- | --- |
|  | Adelhöfer et al. (2019) |  |  |  |  |  |  |  |  |  | Low risk |  |  |
|  | Beeli et al. (2008) |  |  |  |  |  |  |  |  |  | Some concerns |  |  |
|  | Boggio et al. (2008) |  |  |  |  |  |  |  |  |  | High risk |  |  |
|  | Cunillera et al. (2014) |  |  |  |  |  |  |  |  |  |  |  |  |
|  | Cunillera et al. (2016) |  |  |  |  |  |  |  |  | D1 | Randomisation process |  |  |
|  | Friedrich & Beste (2018) |  |  |  |  |  |  |  |  | DS | Bias arising from period and carryover effects |  |  |
|  | Jacobsen et al. (2011) |  |  |  |  |  |  |  |  | D2 | Deviations from the intended interventions |  |  |
|  | Kwon & Kwon (2013) |  |  |  |  |  |  |  |  | D3 | Missing outcome data |  |  |
|  | Lapenta et al. (2012) |  |  |  |  |  |  |  |  | D4 | Measurement of the outcome |  |  |
|  | Leite et al. (2018) |  |  |  |  |  |  |  |  | D5 | Selection of the reported result |  |  |
|  | Li et al. (2019) |  |  |  |  |  |  |  |  |  |  |  |  |
|  | Nejati et al. (2018) |  |  |  |  |  |  |  |  |  |  |  |  |
|  | Nieratschker et al. (2015) |  |  |  |  |  |  |  |  |  |  |  |  |
|  | Osimo et al. (2019) |  |  |  |  |  |  |  |  |  |  |  |  |
|  | Plewnia et al. (2013) |  |  |  |  |  |  |  |  |  |  |  |  |
|  | Sallard et al. (2018) |  |  |  |  |  |  |  |  |  |  |  |  |
|  | Wynn et al. (2019) |  |  |  |  |  |  |  |  |  |  |  |  |
|  | Reinhart & Woodman (2014) |  |  |  |  |  |  |  |  |  |  |  |  |
|  | Silva et al. (2017) |  |  |  |  |  |  |  |  |  |  |  |  |
|  | Yu et al. (2015) |  |  |  |  |  |  |  |  |  |  |  |  |
|  | Schroeder et al. (2022) |  |  |  |  |  |  |  |  |  |  |  |  |
|  | Perotta et al. (2021) |  |  |  |  |  |  |  |  |  |  |  |  |
|  | Vanderhasselt et al. (2020) |  |  |  |  |  |  |  |  |  |  |  |  |
|  | Dormal et al. (2020) |  |  |  |  |  |  |  |  |  |  |  |  |
|  | Mannarelli et al. (2020) |  |  |  |  |  |  |  |  |  |  |  |  |
|  | Fehring et al. (2019) |  |  |  |  |  |  |  |  |  |  |  |  |
|  | Verveer et al. (2021) |  |  |  |  |  |  |  |  |  |  |  |  |
|  | Thunberg et al. (2020) |  |  |  |  |  |  |  |  |  |  |  |  |
|  | Bender et al. (2017) |  |  |  |  |  |  |  |  |  |  |  |  |
|  | Castro-Menses et al. (2016) |  |  |  |  |  |  |  |  |  |  |  |  |
|  | Lau et al. (2019) |  |  |  |  |  |  |  |  |  |  |  |  |
|  | Sedgmond et al. (2019) |  |  |  |  |  |  |  |  |  |  |  |  |
|  | Bashir et al. (2019) |  |  |  |  |  |  |  |  |  |  |  |  |
|  | Bashir et al. (2022) |  |  |  |  |  |  |  |  |  |  |  |  |
|  | Friehs et al. (2021a) |  |  |  |  |  |  |  |  |  |  |  |  |
|  | Smits et al. (2021) |  |  |  |  |  |  |  |  |  |  |  |  |
|  | Boggio et al. (2007) |  |  |  |  |  |  |  |  |  |  |  |  |
|  | Campanella et al. (2017) |  |  |  |  |  |  |  |  |  |  |  |  |
|  | Friehs and Frings (2018) |  |  |  |  |  |  |  |  |  |  |  |  |
|  | Friehs & Frings (2019) |  |  |  |  |  |  |  |  |  |  |  |  |
|  | Stramaccia et al. (2015) |  |  |  |  |  |  |  |  |  |  |  |  |
|  | Stramaccia et al. (2017) |  |  |  |  |  |  |  |  |  |  |  |  |
|  | Sandrini et al. (2020) |  |  |  |  |  |  |  |  |  |  |  |  |
|  | Friehs et al. (2021b) |  |  |  |  |  |  |  |  |  |  |  |  |
|  | Weidler et al. (2022) |  |  |  |  |  |  |  |  |  |  |  |  |
|  | León et al. (2020) |  |  |  |  |  |  |  |  |  |  |  |  |
|  | Chen et al. (2021) |  |  |  |  |  |  |  |  |  |  |  |  |
|  | Bell et al. (2022) |  |  |  |  |  |  |  |  |  |  |  |  |
|  | Campanella et al. (2018) |  |  |  |  |  |  |  |  |  |  |  |  |
|  | Schroeder et al. (2022) |  |  |  |  |  |  |  |  |  |  |  |  |
|  | Dai et al. (2022) |  |  |  |  |  |  |  |  |  |  |  |  |
|  | Poje et al. (2021) |  |  |  |  |  |  |  |  |  |  |  |  |
|  | Brunyé et al. 2021) |  |  |  |  |  |  |  |  |  |  |  |  |

Note. Parallel studies are not eligible for item DS and thus were not rated on that item.

Table 8.

Heterogeneity test SMD

| Moderator Variable |  |  |
| --- | --- | --- |
|  | QE | *p* |
| Null | 165.7620 | *< .0001* |
| Task | 155.5571 | *< .0001* |
| Polarity | 165.3644 | *< .0001* |
| Target Location | 163.0052 | *< .0001* |
| Return Electrode | 162.4166 | *< .0001* |
| Timing | 165.6650 | *< .0001* |
| Current Density | 164.9407 | *< .0001* |
| Duration | 164.9925 | *< .0001* |
| Blinding | 160.6783 | *< .0001* |
| Note. Number of studies = 53, number of effects = 88. QE = Test for Residual Heterogeneity | | |

**Table 9**

Summary of all included studies

| Study | Active \| **Sham** | Study Design | Task | Population | Polarity | Intensity (mA) | Timing | Duration | Target electrode size (cm^2^) | Target | Return | Outcome |
| --- | --- | --- | --- | --- | --- | --- | --- | --- | --- | --- | --- | --- |
|  |  |  |  |  |  |  |  |  |  |  |  |  |
| Boggio (2007) | 12 \| 7 \| **7** | Parallel design | GNG | MDD | anodal | 2 | offline | 20 | 35 | F3 | supraorbital | Anodal stimulation over the left DLPFC showed a significant improvement in number of correct responses compared to occipital lobe stimulation. |
| Campanella (2017) | 15\| **16** | Parallel design | GNG | healthy | anodal | 2 | offline | 20 | 25 | F8 (rIFG) | extracranial | TDCS did not modulate GNGT performance. However, main effect of session. |
| Dai (2022) | 15 \| **14** | Parallel design | GNG | healthy | anodal | 1.5 | offline | 30 | 25 | DLPFC | contralateral supra orbital area | The active tDCS group did not show an improvement in GNGT accuracy after one session. However, an improvement after session 3 was found. |
| Friehs & Frings (2018) | 28 \| **28** | Parallel design | SST | healthy | anodal | 1 | offline | 19 | 9 | F4 | extracranial | Interaction between time and group. The anodal stimulation group showed shorter SSRT post stimulation compared to the sham group. |
| Friehs & Frings (2019) | 22 \| **20** | Parallel design | SST | healthy | cathodal | 1 | offline | 19 | 9 | F4 | extracranial | Interaction between time and group showing an increase SSRT after cathodal stimulation. |
| Stramaccia (2015) | 20 \| 20 \| 20 \| 20 \| **35** | Parallel design | SST | healthy | Anodal, cathodal, anodal, cathodal | 1.5 | offline | 20 | 16 | FC4, F4, F4, FC4 | supraorbital | Anodal stimulation over the rIFG showed lower SSRT compared to sham tDCS. |
| Stramaccia (2017) | 24 \| 23 \| **24** | Parallel design | SST | healthy | anodal | 1.5 | online | 19 | 16 | rIFG | supraorbital | No difference between SSRTs in active and sham tDCS. The cathodal group was excluded due to technical issues. |
| Sandrini (2020) | 15 \| **15** | Parallel design | SST | healthy | anodal | 1.5 | offline | 20 | 25 | rIFG | supraorbital | Anodal tDCS improves SSRT. |
| Sedgmond (2019) | 88 \| **84** | Parallel design | GNG | healthy | anodal | 2 | offline | 20 | 35 | F4 | other brain area | No effect of tDCS on GNGT responses. |
| Bashir (2022) | 23 \| **7** | Parallel design | SST | healthy | anodal | 2 | offline | 20 | 3.14 | C3 (M1 area) | supraorbital | The active stimulation group showed a significant improvement after anodal tDCS compared to sham tDCS. |
| Bashir (2019) | 18 \| **18** | Parallel design | SST | Healthy | anodal | 2 | online | 20 | 3.14 | F3 | C4 | The active group showed improved correct response time during Go trials compared to the sham group. SSRT did not improve. |
| Weidler (2022) | 9 \| **9**  7 \| **9**  5 \| **8** | Parallel design | SST | Alcohol tobacco, healthy | anodal | 1.5 | online | 20 | 35 | F4 | supraorbital | Active groups in alcohol and tobacco user showed improves response inhibition compared to their respective sham groups. |
| Friehs et al. (2021) Study 1 | 15 \| 13 \| **17** | Parallel design | SST | healthy | Anodal, cathodal | 0.5 | offline | 19 | 9 | F4, F8 | F8 rIFG | Anodal tDCS over the rDLPFC and rIFG did not improve response inhibition. |
| Smits (2021) | 46 \| **47** | Parallel design | SST | veterans | anodal | 1.25 | online | 20 | 35 | F8-Cz (rIFG) | T4-Fz DLPFC | Active and sham tDCS did not improve performance during the SST |
| Poje (2021) | 6 \| **4** | Parallel design | GNG | Healthy and Prader-Willi Syndrom | anodal | 2 | offline | 30 | 35 | F4 | Fp1 | Active tDCS resulted in maintanance of reaction time during the GNGT |
| Brunyé (2021) | 24 \| **24** | Parallel design | SST | healthy | anodal | 1.5 | offline | 15 | 35 | F3 | Fp2 | tDCS did not have an effect on response inhibition. |
| Friehs et al. (2021) Study 2 | 23 \| **22** | Parallel design | SST | healthy | anodal | 0.5 | offline | 19 | 9 | F4 | right deltoid muscle | Active tDCS lead to improved response inhibition performance in the post-session compared to pre-session. |
| Leon (2020) | 16 \| **18**  15 \| **12** | Parallel design | SST | Healthy (Man, women) | anodal | 1.5 | offline | 20 | 9 | Fp2 | trapezius | Active tDCS did not improve SST performance more than sham tDCS. |
| Chen (2021) | 30 \| 30 \| **33** | Parallel design | SST | healthy | Anodal, cathodal | 1.5 | offline | 25 | 35 | F4 | Fp1 | Response inhibition improved after anodal and cathodal tDCS compared to sham tDCS. |
| Bell (2022) | 63 \| **63** | Parallel design | SST | healthy | anodal | 1.5 | offline | 20 | 25 | F6 | contralateral supra orbital area | Participants high on sensation-seeking and lack of premeditation showed detrimental effect of tDCS on response inhibition. |
| Campanella (2018) | 18 \| **17** | Parallel design | GNG | healthy | anodal | 2 | offline | 20 | 25 | rIFG | trapezius | Active versus sham tDCS did not false alarms rate. However, active tDCS over the rIFG resulted in a reduction of the drop in accuracy for fast responses. |
| Schroeder (2022) | 20 \| 23 \| **24** | Parallel design | SST | healthy | cathodal | 1 | online | 30 | 1.15 | F3, FC5 | Fp1, Fz, C3, and F7 | Cathodal tDCS over the left IFG and left DLPFC did not modulate response inhibition |
| Adelhöfer (2019) | 19 | Cross-over design | GNG | healthy | anodal | 2 | offline | 15 | 25 | Cz, preSMA | other brain area FpZ | Anodal tDCS did not show an effect on response inhibition |
| Beeli (2008) | 35 | Cross-over design | GNG | healthy | anodal | 1.5 | online | 5.5 | 35 | F4 | extracranial | Anodal tDCS did not show an improvement of response inhibition. Cathodal tDCS show a deterioration of performance. |
| Boggio (2008) | 14 | Cross-over design | GNG | healthy | anodal | 2 | offline | 8 | 35 | other | other brain area | Interaction between tDCS group and sex. Women made less false alarms during anodal tDCS compared to sham tDCS. |
| Cunillera (2014) | 22 | Cross-over design | GNG, SST | healthy | anodal | 1.5 | online | 18 | 9 | rIFG | left IFC | Anodal simulation of the rIFG improved SST performance. |
| Cunillera (2016) | 13 | Cross-over design | SST, GNG | healthy | anodal | 1.5 | online | 20 | 9 | rIFG | other brain area | Differences between active and sham tDCS were non-significant for both the easy and hard SST trials. |
| Friedrich & Beste (2018) | 17 | Cross-over design | GNG | healthy | cathodal | 2 | offline | 15 | 25 | other central | supraorbital | Number of false alarms were significantly higher sham compared to active tDCS. |
| Jacobson (2011) | 11 | Cross-over design | SST | healthy | Anodal, cathodal | 1 | offline | 10 | 25 | rIFG | contralateral OFC, contraleratal IFG | Unilateral anodal tDCS on the rIFG showed a significant improvement compared to sham. |
| Kwon & Kwon (2013) | 40 | Cross-over design | SST | healthy | anodal | 1 | offline | 10 | 35 | M1 (C3 or C4), preSMA (Cz) | supraorbital | Response inhibition improved significantly in the pre-SMA condition compared to M1 and sham tDCS. |
| Lapenta (2012) | 28 | Cross-over design | GNG | healthy | Anodal, cathodal | 1 | online | 14 | 35 | T3 and T4 | extracranial | No effect of tDCS on response inhibition. |
| Leite (2018) | 16 | Cross-over design | GNG | healthy | Anodal (uni- and bihemispheric) | 1 | offline | 30 | 35 | rIFG | contralateral | No effect of tDCS on response inhibition. |
| Li (2019) | 24 | Cross-over design | SST | healthy | Anodal, cathodal | 2 | online | 18 | 16 | rIFG | extracranial | Active tDCS, compared to sham tDCS, results in significantly better performance. |
| Nejati (2018) | 24 | Cross-over design | GNG | healthy | Anodal, cathodal | 1.5 | online | 20 | 35 | F3 | Fp2 | Post-hoc test revealed a significant improvement in response inhibition in the active compared to sham tDCS group. |
| Nieratschker (2015) | 41 | Cross-over design | GNG | healthy | cathodal | 1 | online | 20 | 35 | F3 | supraorbital right | Interaction between COMT polymorphism and tDCS condition. Cathodal tDCS impaired response inhibition in the Val-Val carriers compared to Met carriers. |
| Osimo (2019) | 51 | Cross-over design | GNG | other patients | Anodal, cathodal | 1.5 | offline | 20 | 25 | F4 | extracranial | No effect of anodal or cathodal tDCS on response inhibition accuracy. |
| Plewnia (2013) | 46 | Cross-over design | GNG | healthy | anodal | 1 | online | 20 | 35 | F3 | supraorbital | No effect of tDCS on response inhibition. |
| Sallard (2018) | 16 | Cross-over design | GNG | healthy | anodal | 1.5 | offline | 20 | 35 | rIFG | supraorbital | No difference in false alarms between active and sham tDCS. |
| Wynn (2019) | 26 | Cross-over design | GNG | healthy | cathodal | 2 | online | 30 | 35 | other | extracranial | Number of false alarms was significantly lower in active compared to sham tDCS. |
| Reinhart & Woodman (2014) | 18 | Cross-over design | SST | healthy | Anodal, cathodal | 1.5 | offline | 20 | 16 | FCz | left cheek | Anodal group showed reduced error rates and cathodal showed increased error rates compared to the sham group. |
| Silva (2017) | 35 | Cross-over design | GNG | fibromyalgia patients | anodal | 1 | online | 20 | 35 | F3 | supraorbital | No effect of tDCS on false alarms. |
| Yu (2015) | 23 | Cross-over design | SST | healthy | anodal | 2 | offline | 20 | 16 | preSMA | left cheek | SSRT was significantly lower in active compared to sham tDCS. |
| Schroeder (2023) | 14 (restrained) and 15 (unrestrained) | Cross-over design | SST | Healthy (restrained vs. unrestrained eaters) | anodal | 1 | online | 20 | 3.1416 | FC4 (rIFG) | contralateral arm | Interaction between group and tDCS. Restrained eaters showed longer SSRTs during sham tDCS. But restrained and unrestrained eaters performed similarly under anodal tDCS. |
| Perrotta (2021) | 12 | Cross-over design | GNG | healthy | anodal | 1.5 | offline | 18 | 9 | T4-Fz F8-Cz | T3-Fz F7-Cz, left IFG | No effect of tDCS on response inhibition. |
| Vanderhasselt (2020) | 38 | Cross-over design | GNG | healthy | anodal | 2 | offline | 20 | 35 | F4 | F3, DLPFC | Three-way interaction between tDCS, reward, stimulus type. The effect of tDCS was significant in reward go trials. |
| Dormal (2020) | 20 | Cross-over design | GNG | Alcohol, healthy | anodal | 1.5 | online | 20 | 35 | F3 | Fp2 | No effect of tDCS on false alarms in neither group. |
| Manarelli (2020) | 8 | Cross-over design | GNG | healthy | cathodal | 2 | offline | 20 | 25 | right cerebellar cortex | right deltoid muscle | Pre-tDCS difference in false alarms between groups was significant. Post-tDCS difference in false alarms was not significant. |
| Fehring (2019) | 73 | Cross-over design | SST | healthy | anodal | 1.5 | offline | 10 | 10 | L DLPFC | supraorbital | No difference between active and sham tDCS after the first timepoint. |
| Verveer (2021) | 22 | Cross-over design | GNG | healthy | anodal | 1.5 |  | 20 | 0.25 | Fz (dACC) | Fp1, Fp2, F7, F8 | No effect of HD-tDCS on GNG performance. |
| Thunberg (2020) | 18 | Cross-over design | SST | healthy | anodal | 2 | online | 20 | 25 | IFG FC7 and FC8 | CP8 and P8 and CP7 and P7, left IFG | No effect of tDCS on Stop SST performance. |
| Bender (2017) | 18 | Cross-over design | SST | healthy | Anodal, cathodal | 1 | offline | 9 | 25 | preSMA | extracranial | No effect of tDCS on SST performance. |
| Castro-Meneses (2016) | 14 | Cross-over design | SST | healthy | anodal | 1.5 | online | 15 | 25 | rIFG | left cheek | No effect of single session tDCS on SSRT. |
| Lau (2019) | 10 | Cross-over design | GNG | Parkinson | anodal | 2 | online | 20 | 35 | left DLPFC | supraorbital | No effect of tDCS on false alarms in Parkinson patients. |

Note. Sample sizes in bold indicate the sham group.
